# Healthcare professionals’ experiences and perceptions regarding health care of indigenous pregnant women in Ecuador

**DOI:** 10.1186/s12884-022-04432-5

**Published:** 2022-02-04

**Authors:** Tannia Valeria Carpio-Arias, Nervo Verdezoto, Marta Guijarro-Garvi, Victoria Abril-Ulloa, Nicola Mackintosh, Parisa Eslambolchilar, María Teresa Ruíz-Cantero

**Affiliations:** 1 Research Group on Food and Human Nutrition (GIANH), Faculty of Public Health, Higher Polytechnic School of Chimborazo, Pan-American Sur Km 1 1/2, Riobamba, Ecuador; 2 School of Computer Science and Informatics, Human-centered Computing Group, Cardiff University, Cardiff, UK; 3 Department of Economics, University of Cantabria, Cantabria, Spain; 4 Public Health Research Group, University of Alicante, Alicante, Spain; 5 Research Group on Public Health, Nutrition and Physical Activity in the Life Cycle, University of Cuenca, Cuenca, Ecuador; 6 Department of Health Sciences, University of Leicester, Leicester, UK; 7 Public Health Research Group, University of Alicante, Alicante, Spain; 8 CIBERESP, Madrid, Spain

**Keywords:** Health care professionals, Prenatal care, Gender, Human rights, Pregnant women, Ethic minorities, Indigenous communities

## Abstract

**Background:**

Pregnancy is an important life experience that requires uniquely tailored approach to health care. The socio-cultural care practices of indigenous pregnant women (IPW) are passed along the maternal line with respect to identity, worldview and nature. The cultural differences between non-indigenous healthcare professionals (HPs) and IPW could present a great challenge in women’s health care. This article presents an analysis from a human rights and gender perspective of this potential cultural divide that could affect the health of the IPW in an Andean region of Ecuador with the objective of describing the health challenges of IPWs as rights holders through the experiences and perceptions of HP as guarantors of rights.

**Methods:**

We conducted 15 in-depth interviews with HPs who care for IPW in Chimborazo, Pichincha provinces of Ecuador. We utilized a semi-structured interview guide including questions about the experiences and perceptions of HPs in delivering health care to IPW. The interviews were recorded, transcribed and subjected to thematic analysis in Spanish and translated for reporting.

**Results:**

We found disagreements and discrepancies in the Ecuadorian health service that led to the ignorance of indigenous cultural values. Common characteristics among the indigenous population such as illiteracy, low income and the age of pregnancy are important challenges for the health system. The gender approach highlights the enormous challenges: machismo, gender stereotypes and communication problems that IPWs face in accessing quality healthcare.

**Conclusions:**

Understanding the diverse perspectives of IPW, acknowledging their human rights particularly those related to gender, has the potential to lead to more comprehensive and respectful health care delivery in Ecuador. Further, recognizing there is a gender and power differential between the provider and the IPW can lead to improvements in the quality of health care delivery and reproductive, maternal and child health outcomes.

## Background

In the health care arena disparities have been observed in universal access to health care services and these disparities are especially common in low- and middle-income countries [1] such as Ecuador. Inequalities are higher amongst women than men [2, 3] and that leads to further disadvantage in health care related to poverty and the vulnerability of indigenous women during pregnancy (Indigenous pregnant women -IPW) [4, 5]. This is the case of the Chimborazo and Pichincha regions, where there are indigenous communities who have some of the highest maternal mortality rates in the country (37 per 100.000 and 59.5 / 100.000 - 9.5 / 100.000 life births respectively) [6, 7]. Many Latin American countries like Ecuador are characterized by having a multiethnic and multicultural population [8, 9]. In Ecuador, approximately 25% of the population is indigenous, 10% Afro-descendant, 10% Caucasian, 5% of other ethnic origin, and 50% mestizo [10, 11] Mestizos, who have indigenous and Spanish heritages, have different identities and practices from those of indigenous people and, in general, have greater power over these groups [10]. Furthermore it is known from the literature that successive forms of discrimination against the indigenous population have persisted from colonial times to the present day [11]. Previous studies from around the world show that cultural issues related to prenatal care in can lead to delayed diagnosis of problematic health issues. These include service delivery problems [12] and delayed access to prenatal care [13, 14]. Furthermore, low access to prenatal care has been linked to poverty [15, 16] and low levels of education [17]. There is also limited information on implementation of inter or cross cultural approaches to health care [18] with a little documentation on alternative or traditional medicines (a health care approach outside of conventional or conventional western medicine) and even less data on specific indigenous medical practices [19]. Additionally the paucity of data for indigenous groups shows that comparisons by ethnic groups can be problematic [5, 10]. Health services in Ecuador have not yet properly adapted an intercultural approach despite an improvement in the provision of services in the last few decades [20, 21]. The western health care system tends to ignore the need for alternative and indigenous care and prevents women from putting into practice the rituals and practices that are needed for help define own identity [20–22]. The World Health Organization (WHO) recognizes lack of access, delayed used and poor quality of care as determinants of inequalities in health [23, 24]. Incorporating a human rights perspective into the analysis of reproductive and maternal care of IPW could be useful to avoid or reduce gender biases in health care and broaden the context of health to be one that is more holistic [25]. This human rights approach focuses on the fulfillment of health care, including the availability, accessibility, acceptability, quality and relevance of care [24]. The human rights perspective interacts with the gender perspective, which is interested in how women individually make decisions related to their health in a limited range of options derived from gender relations [26]. Use a human right and gender informed lens could reduce the tension between individual practice and social structures [12, 16, 25]. In addition, IPW should be considered as an autonomous actor with the agency to make decisions based on her own culture and preferences [3]. Discrepancies have been observed between the support that IPWs want and the support that the health system can provide [27] framed as predominantly mestizo healthcare [21]. The combining of a human rights and gendered approach has demonstrated favorable health outcomes for the IPW [28]. The misalignment of health systems with the cultural needs of IPW are risk factors for poor prenatal and maternal care. This article aims to describe the health care challenges of IPWs as human rights holders through the experiences and perceptions of health professionals (HPs) as guarantors of rights.

## Methods

Based on our studies on maternal and child health, an article has been published with results regarding the barriers faced by pregnant indigenous women in rural areas of Ecuador when seeking medical attention. In this qualitative study we used a semi-structured interview guide with HPs [29] in the care of IPW in the Andean region of Ecuador. Two researchers (V.C-A and V.A-U) with experience in health sciences and qualitative studies conducted the interviews. The design and analysis of the study was carried out by a multidisciplinary team of experts in public health, human-computer interaction and social sciences applied to health.

### Participants

HPs participants were purposively selected with previous experience in caring for IPW. HPs who work in the provinces of Chimborazo (*n*=11) were invited to participate, as it is the province with the largest indigenous population in Ecuador [6]. HPs from the province of Pichincha (*n*=4) were also included to complement the initial interviews. All HPs at the time of the study were working in hospitals in Ecuador. Participants (*n*=15) were all HPs, mestizo and no one HPs talks indigenous languages. They worked in public facilities (*n* = 11 Public Health System; *n* = 4 social security system). Participant´s age range between 26-39 years old, gender (female 10, male 5) and had diverse professional backgrounds: nine specialist physicians (four gynecologists, four family doctors, and one imaging specialist), four general doctors, and two nurses. We did not interview any midwives.

### Ethics approval and consent to participate

Eligible participants received a participant information sheet and a consent form by email. All participants received an explanation of the study and signed the informed consent. A copy of the informed consent (signed) was delivered in person or emailed to the researchers. This study was performed in accordance with the Declaration of Helsinki. Ethical approval for this study was granted by the ethics committee at Cardiff School of Computer Science and Informatics as there was no local ethics committee at ESPOCH University. But letters of support were received from local partners: National Council of Rural Parish Governments of Ecuador (CONAGOPARE).

### Recruitment

The participants were recruited between February and June 2020. Initially, the indigenous leaders of the province of Chimborazo were contacted through the CONAGOPARE who put the researchers in contact with HPs who work in areas with a high prevalence of indigenous population. Participants were contacted by phone first and they were informed about the study. Participants were informed that the researchers worked at local universities. A date and time for the interview was scheduled for HPs interested in participating. Snowball sampling was used until we reached saturation through the interviews carried out [30]. A total of 17 HPs were contacted of which two refused to participate due to lack of time.

### Data collection

Interviews were conducted in person (*n*=5; in the CONAGOPARE meeting room in the province of Chimborazo) on February 2020 or by video call (n=10; researchers and HP were at home) on March-June 2020. Only one researcher and the HPs were present during the interviews. The interviews had an average duration of 38 minutes.

A semi-structured interview guide was used, considering five categories (Table 1) that includes characterization of patients, HPs work practices, cultural relation and communication perceptions of characteristics of IPW and health care infrastructure. Questions in every category, based on a human rights and gender perspective, present the researchers' interest in capturing the cultural similarities and differences between Mestizo HPs and IPW. Furthermore, we present in the results section the main findings between the perspectives of male health professionals and female health professionals when differences were found according to these two groups.

**Table 1:** Topic guide for the interviews with health professionals about health care in indigenous pregnant women

Categories	Topics
Characteristics of indigenous pregnant women	• Cultural characteristics. • Socio-demographic characteristics
Professional and personal training to meet the challenges of healthcare at IPW	• Training or education to care IPW.
Communication	• Similarities and differences between mestizo HPs and IPW related to communication themselves. • Mother language and second language for interaction between HPs and IPW.
HPs Work description	• Most important activities • Work responsibilities: Institutional or national laws that the HPs perceives to be positive or negative for their work
Characteristics of human, physical and technological equipment for the care of pregnant indigenous women	• Physical and human infrastructure of hospitals and / or health centers • Equipment, supplies, physical space. • Use of technology: types, utility.

### Data management and analysis

General notes were taken: interview number, interview location, start and end time. The interviews were audio-recorded, transcribed verbatim in the HPs’ language (Spanish) and anonymized to protect the identity of the participants and maintain confidentiality.

Themes and subthemes emerged from the analysis of the proposed categories. We used the MS Word processor to mark the themes and subthemes for coding with different colors. V.C-A and V.A-U met regularly during the data collection period to discuss emerging issues or controversies. The information review protocol consisted of each researcher independently reading and coding a subset of interview transcripts to obtain a set of themes. Researchers MT. R-C and N. V, evaluated the cases and discussed further with V.C-A and V.A-U, in case of differing interpretations. To reflect on the cases and recognize the positionality of the researchers as observers.

The data was analyzed using an inductive approach [31]. We used a thematic network analysis, refining the basic and organizational themes to reach the global ones (opportunities and barriers) [32]. Text segments were presented under key themes and organizational themes, and compared for meaning and identifying similarities, and differences [32]. The thematic analysis was checked several times by researchers V, C-A and V. A-U and the themes were derived from the available data. The thematic analysis was sent by email twice to a population subgroup for review. Comments or suggestions were mostly linguistic in nature, while content changes were added to the thematic analysis. Finally, the report was translated to English by the Spanish-speaking researchers to share with all non-Spanish speaking authors to comment on the last iteration of themes.

## Results

From the information collected, four themes emerged on the perception and experiences of HPs related to the values, practical behaviors or opinions of indigenous pregnant women:

### Home versus hospital and health care models

The public health system in Ecuador includes home health care in rural areas, which are predominantly home to indigenous communities. HPs identified this aspect as positive, which makes women accept hospital care when necessary

*"Health care at home has made it possible to provide health care to women who do not go to health centers by themselves. In some cases, we have identified maternal complications such as pre-eclampsia or gestational diabetes and we have convinced women to go to a hospital " [Woman, Medical Gynecologist-Province of Chimborazo, 37 years].*

However, home visits could have shortcomings in terms of care provision such as lack of access to physical resources such as imaging tests or biochemical tests. The coordination of the health system must provide for this aspect. Especially women in rural areas might decide only to receive visits rather than visiting the healthcare settings or centers

*“There are women whose only care is home visits, but specific tests cannot be performed on them. Without test results like hemoglobin, care is complicated.” [Woman, Family doctor, Chimborazo Province, 38 years]*

Furthermore, HPs identified home birth as a dangerous situation for a mother and her baby, since complications that arise cannot be resolved promptly.

*“Home births attended by midwives, there are many of them that work well, but there are others that can be complicated and the patient dies. [Woman, Obstetrician gynecologist, Pichincha province, 35 years].*

But, on the other hand, hospital birth could affect women's identity in different ways, as there are a variety of gender norms on how women should and shouldn't give birth. HPs recognized some preferences in the birth include and overcoming the birth pain of IPW. Such as being with her family (especially female members), vertical childbirth, or drinking herbal teas. However, the healthcare system does not allow women to make these decisions. This could explain by rigid hospital protocols and because it is possible that there was little capacity within the system to adapt to the women´s preferences. Nor is the gender perspective considered with regard to pain management during labor.

*“Women think (or know) that the hospital does not allow them to be comfortable and with company, or to be with their clothing or their waters (infusions) for childbirth” [Obstetrician gynecologist, Pichincha province]”.*

The exercise of human rights for inclusive care deserves the adaptation of cultural practices. The dominant mestizo culture influences health care delivery models that may not align with the needs of IPW generating mistrust in of HP and their facilities.

*"Indigenous women have (their own) practices such as burying or saving the placenta ... there are always preferences to give birth at home" [Woman, General practitioner, Chimborazo province, 35 years]*

The lack of training in indigenous customs could raise issues for HPs and the care they offer to IPW it can create tension without mutual understanding. Intercultural adapted childbirth, promoted by the Ecuadorian Ministry of Public Health, tried to use elements according to the culture of each person, as long as this does not interfere with the medical safety of a patient. But it was difficult for HPs to apply methods of care to childbirth in the indigenous way, such as attend a vertical birth when they do not know how to care for women in this position.

*“I could not help in a vertical birth; I do not know it and I do not think I can train myself. It may be that some infusion (or tea) of the ones that is used to take is good, and others very bad, but I do not know either”* [Woman, Nurse, Pichincha province, 26 years]

But in general HPs weren't sure if herbal teas are good or bad for women's health.

In particular cases, such as drinking infusions with alcohol, concerns and specific consequences were mentioned.

*“I respect customs. But ingesting herbal teas, such as cinnamon water mixed with artisanal alcoholic beverages can cause very intense uterine contractions, it is very dangerous” (Man, Family doctor, Chimborazo province, 38 years].*

Working with human resources who for example a cultural broker (someone who understands both cultures and can explain the rationale for each) can be trusted by IPW, is a strength for the development of antenatal and postnatal care for culturally diverse women. HPs are aware of the difficulty in providing health services to IPW based in desired indigenous customs in the context of their western hospitals or western training. In this study, HPs knew that they are not popular among indigenous women and reported that pregnant women preferred the care of “midwives” to a hospital or health centre, especially during childbirth.

*"It is very common for women to give birth with their midwife, it is better accepted than coming to the hospital". [Man, General practitioner, Chimborazo province, 35 years].*

### Challenges of the health system related to sociodemographic characteristics of IPW

Certain sociodemographic characteristics amongst IPWs were found to hamper their medical care. Firstly, illiteracy and low educational level have been identified by HPs as a great challenge when giving instructions, prescriptions and health and care indications to IPW.

*“I am surprised to have found, for example, an illiterate 29-year-old indigenous woman, which makes it difficult to give written instructions on medications, quantities, schedules or health care instructions” [*Woman, Family doctor, Chimborazo province, 35 years*]*

Secondly, the low-income level amongst IPW represents a problem for their health care. HPs reported this situation as a serious limitation. Furthermore, it is a failure of the Ecuadorian public health system not to always have, at all times, basic medicines for pregnant women.

*“Sometimes we don't have the medicines in the health center and we send them to buy, but they don't even have money enough to eat”* [Woman, Nurse, Pichincha province, 26 years]

Thirdly, specialized hospitals are only based in three large cities in the country and access to such hospitals has been identified as a limitation, i.e., travelling to such facilities for IPW is difficult due to several reasons:

*"Travel can be a problem for indigenous women, because of the cost of transportation. Also, children, animals and crops cannot be abandoned."* [Woman, Family doctor, Chimborazo province, 38 years].

Finally, another challenge described by HPs was the age of pregnant women. It was described that there are both indigenous adolescent women and indigenous women of advanced age to be pregnant. The care needs of an adolescent pregnant woman or of a woman of advanced age to be pregnant could demand greater care and may have a greater risk.

*"The health care we provide must be both for adolescents or girls and for adult women who already have risks to their health due to pregnancy"[Woman, Gynecologist. Chimborazo Province, 37 years].*

### Gender issues and health care for IPW

In addition to women's willingness to give birth at home, which is strongly resisted by HPs (as discussed above), there are gender stereotypes. Our findings revealed how the gendered livelihood system around indigenous communities was about men’s control over every aspect of women’s lives, and as a consequence of ‘machismo’, (that emphasizes men’s dominance while encouraging women’s submission [3]), women did not want to go to the antenatal care alone. Regarding the “machismo” context, professionals of both sexes are aware of the machismo context and its influence in achieving the highest possible level of health for indigenous women through health care. Although, all professional women; that is, 10, and only 3 men make explicit (or indicate) that the IPW do not want to go to the consultations, do not attend routine check-ups, and do not make any decision without the presence or consent of the husband.

*"IPW do not want to go to the consultations, because of the husbands' machismo, since it is considered that when the doctor values it, it is as if they were violating their privacy as a woman. Women do not make any decisions without the consent of the husband" [Man, Family doctor. Chimborazo Province, 35 years] “ also the husband will not let to use contraceptive methods” [Woman, General Doctor, Pichincha province, 29 years]*

*“The most “machismo” part that exists in my opinion is that every woman has the right to choose what she wants to do with her body, what happens, that women sometimes give birth to their fifth child, you give them family planning counseling and you tell her that sterilization is best, that a sixth - seventh pregnancy is very risky for them or if she is in a caesarean birth, a third or fourth is too risky. Many of them do not make the decision for them, which should be a decision of her own, of the woman because you are the one carrying the pregnancy, but they have to corroborate the decision with their husbands and many times their husbands are so “machismo” that they do not want to they hook up. Women are afraid of their husbands, I think they beat and mistreat them, your partner's opinion is fine, but in the end the decision is yours” [*Woman- Obstetrician Gynecologist-Pichincha province, 35 years*].*

*“It is a conflict to attend to indigenous women because of machismo, and the bad thing is that they do not always go to the consultation with their husbands, and they should, they would save us time” [*Woman-Gynecologist-Pichincha province, 36 years*]*. “*The customs are fine, we all have one, but the problem when the decisions of the spouses come into play ... as health professionals we realize that it is a bit exasperating because women remain silent and tell us that they are going to come back and we already know that it is because first, they are going to ask the husband” [*Woman-Gynecologist-Pichincha province, 35 years*]. The beliefs in the communities are strong, they are macho and their beliefs are not sure if they help or harm [*Man-Family doctor-Chimborazo province, 38 years*]*. *The beliefs in the communities are strong, they are machismo and their beliefs are not sure if they help or harm [*Man-Family doctor-Chimborazo province, 38 years*]*

*If there is no approval of the family (of the woman or of the couple), as well as the approval of the couple and in certain cases even of the community, then there is no way to take any action, not even to save their life, we will see them almost dead or the fetus dead and they do not want to go to the hospital because the husband does not want to. [*Man. General practitioner-Chimborazo, 35 years*]*

Furthermore, decisions about prenatal care for IPW seem to depend on what has been passed down in her family or family tradition. HPs reported that women have few prenatal visits and that the women went to the health centre only for childbirth or when complications occurred.

*“Women take their grandmothers as an example, saying that they never went to the doctor and nothing happened to them, they do not know about the dangers for them and their babies" [*Man, *Family doctor, Chimborazo province, ]*

HPs perceived that IPW tend to be introvert sand that this was not the case with indigenous men. Shyness and shame about receiving care was linked to reluctance to share aspects of their life such as their relationship or the presence of risk factors such as vaginal secretions, ulcers, or bleeding. In addition, HPs reported how indigenous women resisted the physical examination. Some HPs even felt that IPW do not volunteer information when they attend IPW. Although HPs are not sure of the reason, they speculate it could be related to shyness amongst IPW:

*"Being an IPW we are more careful because they do not like to tell us the truth, they are very shy and ashamed" [Men-*Imaging physician, Chimborazo province*, 38 years].*

All the professional men and half of the health professional women are aware that the cultural reality of IPW governs communication during health care, insofar as they do not transmit all the information “they are silent”, "They are ashamed " they do not tell the truth "

*“Yes, the difficulties would come to be above all that focuses on culture, I mean indigenous women, in terms of their modesty it is to some extent the barrier that prevents us from managing them efficiently, this would be one of the biggest drawbacks, the modesty leads to them not telling us everything that happens to them and that complicates things”. [Man-Family doctor-Chimborazo province, 38 years].*

*“Indigenous women are very modest. They lie to us a lot, especially when they want to hide a pregnancy [Woman- Obstetrics Gynaecology-Pichincha province, 35 years].” It seems to me that there are things that they do not tell us, especially to cover the husband, so as not to make him look bad” [Woman-nurse-Chimborazo, 26 years s].*

HPs reported challenges in overcoming IPWs fears, particularly when reinforced by family and peers. This put HPs in a vulnerable position in terms of accountability for their safety.

*“I had a pregnant teenager. I went to her house to see her and I asked her if we could go to the Health Center to take her blood pressure because I did not have the blood pressure monitor there. She didn't want to go and when she finally agreed her aunt told her, why are you going to go (to the health center) if they don’t give you any medicine? The teenager refused again. In that case, if the woman died, that would be our fault*” [Woman, Obstetrician gynecologist, Pichincha province, 35 years]

### Structural challenges and opportunities for health care

First, both the most basic health centers (care level 1 in Ecuador) and hospitals with specialized levels of care (care level 3 in Ecuador) have what is necessary for prenatal care. However, the HPs in this study noted the differences between the provisions of public and private hospitals, especially the lack of space or privacy in the former.

*"The Ministry of Public Health establishes that each health center has a basic kit to care for pregnant women in emergencies ... but each kit that we use must be reported" "It is not the same to care for a patient in a room with 20 beds than in a private room”* [Woman, Family doctor Pichincha province, 35 years]

Secondly, according to the experiences of the HPs interviewed in this study, COVID-19 has represented an important development in the use of social networks, also with the indigenous community. During COVID outbreak, HPs gave their phone number to patients and communicated via WhatsApp. This was helpful for communication and prenatal care instructions could be given through this way.

*"In the emergency due to COVID-19, WhatsApp helped a lot" [Woman, Obstetrician gynecologist, Pichincha province, 35 years]”*

On the use of technology, it not only serves HPs but also mothers.

*"A simple and friendly application that provides accessory and alternatives to women who cannot read, for example, and that allows them to identify alarm signals in case of complications, risk of abortion, maternal death and medical procedures*"[Woman, Obstetrician gynecologist, Pichincha province, 35 years]

But communication between HPs and IPW is challenging. On the one hand, IPW from the Andean area of Ecuador speak Quechua (language commonly spoken in the Chimborazo province) and Spanish. While HPs speak only Spanish and there are no interpreters if the IPW speak only Quechua. On the other hand, HPs perceive that IPW do not usually ask questions. Even the HPs mention that the IPW misunderstood the message. The reason for not asking was not clear. Although in other questions it is mentioned that IPW is shy. We found that no one among HPs questions their lack in intercultural language or inability to communicate with their patients.

We found that no one among HPs questions their lack in intercultural language or inability to communicate with their patients.

*"With indigenous women we seek to make communication clearer and simpler, we look for words or colloquial sayings without losing meaning, ... indigenous women ... if they did not understand something, they simply do not ask again"* [Man, General Practitioner, Chimborazo province, 35 years].

But they are not trained to develop their professional skills in other cultures.

*“I do not know any indigenous doctor who works in the area, most of us are mestizo, it is difficult to ask for help to understand the language or culture of patients”* [Man, General Practitioner, Chimborazo province, 35 years].

Another structural challenges was that HPs were also distressed by the prospect of being prosecuted by the Ecuadorian justice system if a pregnant woman dies in circumstances that are not directly related to their professional practice [33]. This is a policy designed to prevent maternal mortality but seems like an undue burden on the provider. This was mentioned by the HPs in the interviews and reported as one of their major frustrations at work:

*"Nowadays: if a woman does not take her medication or does not attend her check-ups, and dies, it is our fault, and we can be imprisoned for that. That always worries all health professionals*" [Woman, Obstetrician gynecologist, Pichincha province, 35 years]

HPs in this study had identified that IPW may have beliefs around contraceptives related to side effects (cancer or other diseases). It also highlights that IPWs are disempowered in their decisions about their own body and health care.

*"We propose to do the ligation and they say: “they told me that if I have the ligation, I will get a bad temper and my abdomen will hurt " [Man, General Doctor, Pichincha province, 35 years]*

Moreover, HPs reported that IPW refused to take contraceptives and other medications including nutritional supplements.

*"Many women think that the doctors are going to harm them, or that the nutritional supplements are going to cause the baby to grow too much and that this represents problems in the birth …it is frustrating to know that the supplements or medicines are discarded to the pigs”* [Woman, Family doctor, Chimborazo Province, 36 years].

*“During the home visits we have found intact packaging of the nutritional supplements. Vitamin or mineral capsules that women have not taken”* [Man, Family doctor, Chimborazo Province, 35 years]

## Discussion

Quality reproductive health, within the framework of human rights and gender equality for IPW, still requires work in the Andean regions of Ecuador where most of its inhabitants belong to indigenous communities. The results of this study show the needs of the IPW regarding their health care from the perspective of HPs. We identified four content profiles that highlight the tension experienced between a predominantly mestizo conventional care model, which displaces alternative and indigenous health care. We found disagreements in the Ecuadorian health care service leading to indigenous cultural values being ignored. We found discrepancies in the care models of the formal system versus traditional care that can be given at home. Common characteristics among the indigenous population such as illiteracy, low income and the age of pregnancy are important challenges for the health system. In addition, the gender approach highlights the enormous challenges that IPW face in accessing quality health care, these challenges are: machismo, gender stereotypes and communication problems. Finally, we presented list structural challenges of the health system and possible digital alternatives for its improvement. Our study may also be useful in other indigenous populations of Ecuador, such as those that inhabit the Amazon and in other countries with diverse ethnic populations and in intercultural care to improve their inclusion in health systems.

There is a cosmovision among indigenous peoples that characterizes their beliefs, values and desires and this is accentuated during pregnancy [34]. HPs expressed concerns about certain practices associated with customs of IPW: infusions with alcohol, for example, could cause strong uterine contractions and increase the risk of preterm birth [35] so they should be monitored.

Other practices such as the preference to give birth at home show a disarticulation between what HPs and IPW want. Giving birth at home for the HPs interviewed in this study is not an option, the fear of complications is consistent with the world statistics of maternal mortality [7, 36, 37] and adverse perinatal outcomes [38] which in the vast majority of cases could be easily avoided in a hospital [39, 40]. However there is evidence that in some countries like the USA, deliveries can be safe as long as the midwife is qualified or certified [41]. Considering that Ecuador is a territory with diverse indigenous peoples, whose existence is recognised in the Constitution, in the exercise of human rights that favour indigenous people and gender equality. Public health and HPs services could strive to move towards more inclusive care models that may include a kind of cultural intermediary, for example, a woman present from their culture who is able to translate and mentor women to improve confidence, reduce tension with the supplier and incorporate the different meanings and worldviews of indigenous peoples [42].

In addition, the uncomfortable nature of public hospitals and the restriction of cultural customs make giving birth at home a priority for IPW. Giving birth in a hospital, when it is preferred (and possible) to home-birth negates respect for the woman's decisions, and could increase her distrust and disagreement with the conventional health system. It was found in this study that women tend to differentiate between the value and the benefit of their health situation that leads them to seek help in the hospital but only in cases of emergency or complications, which is consistent with the practices of women in South Africa [43] and in the Ecuadorian Amazon [44].

Other studies have already documented that the lack of cultural considerations may be related to wellbeing issues amongst indigenous people [18, 45] and that culturally accepted medical practices provide better results [46]. Therefore, if the identity of an IPW and her beliefs and values are not respected within the health system, the already limited range of decisions that they have is further restricted.

One of these limitations is machismo and gender stereotypes (view or preconception about attributes or characteristics, or the roles that are or ought to be possessed by, or performed by, women and men) [47], which could lead to little cooperation between women and the HPs and refusal of family planning options such as birth controls by the couple. In some cases, the delay in providing health care were linked the HPs being a man and the IPW’s partner not allowing her to attend the consultation. In other cases, when the woman's health decisions depended on the husband e.g., family planning methods, delays or refusal by the husband was reported. Machismo and gender stereotypes have been documented as strongly ingrained characteristics of Ecuadorian indigenous culture [21, 48]. And it has also been observed that women are more accepting of contraceptive methods than men [49] so empowering women can be the key to avoid unwanted pregnancies [21].

But in addition, the delay in the search for medical attention in indigenous populations has been explained based on the lack of confidence that seeking care from a health professional will change health outcomes [11, 50, 51]. Therefore, it is urgent to implement a more inclusive health system that guarantees empowerment in the decisions and well-being of the pregnant woman [25, 39]

Other limitations are related to sociodemographic problems: low income and problems to travel to the hospital (due to distance and cost) both to give birth and to receive care and buy medicines. The challenges identified in our research are not limited to Ecuador and they could extend to other countries [8] for example, rising cases of maternal death and discriminatory behavior in the health service towards ethnic minorities [11] are noted globally. The incidence of poverty in Ecuador in 2019 amongst ethnic group was 20.3% for mestizos, 43.5% for montubios and 51.5% for indigenous people [52]. In addition, low income has been considered an indicator for inequality in maternal and child health care in populations such as Canada [53] and Nigeria [54]. Low income also contributes to transportation issues and purchase of medicines [55] and other problems like unsafe infant sleep practices [56].

Finally, low level of education could relation with communication barriers, indigenous people have (20.4%), compared to mestizos (5.1%) in Ecuador [7] with greater inequalities in women versus men (7.7% and 5.8% respectively). The low education level also contributes to misunderstanding HPs instructions and or prescriptions. Although Quechua is another native language of the Andean indigenous population in Ecuador and the intercultural education system advocates the education in the native language, the mestizo knowledge of Quechua is scarce [21].

## Conclusions

Beyond macrostructural factors such as democracy [57] or governance [58], the challenges associated with the quality of IPW health care in Ecuador are linked to beliefs, values, and customs of its indigenous communities, largely unknown by the conventional health system, which is run by mestizo HPs.

Health care decisions and needs that are culturally best suited to IPW are a human right of this population group. However, gender inequality could be a challenge in the formulation of public policies that take care of IPW. Finally, structural and socio-demographic challenges such as low education and low income must be considered in comprehensive protection plans for vulnerable groups such as IPW.

HPs need an IPW culture-respectful approach to exercising reproductive health rights and gender equality. Understanding what works and how to improve IPW health care is imperative if countries like Ecuador want to advance health equality, from the systems level to the individual level of health care, beyond their policies and initiatives on paper [46].

### Strengths and limitations

The main strengths include the focus on a little-explored topic. Ethnic minorities around the world require special considerations to improve their inclusion in health systems and thus improve their quality of life [59].

On the other hand, it is important to note that this paper builds on our previous engagement with indigenous pregnant women [21], and here we only reported information from the HPs’ perspectives and in two particular provinces of Ecuador. Further research needs to consider pregnant women and their indigenous families and healthcare professional in other provinces in Ecuador. In addition, ethnic groups from Andean region may present different characteristics, so it is suggested to carry out similar studies with other ethnic groups.

## Data Availability

Anonymous interview transcripts will be shared upon receiving reasonable request. Please contact the corresponding author through this email, tannia.carpio@espoch.edu.ec

## Abbreviations

HPs: Health professionals
IPW: Indigenous pregnant women
CONAGOPARE: National Council of Rural Parish Governments of Ecuador
WHO: World Health Organization

## References

[1] Lawn JE, Blencowe H, Waiswa P, Amouzou A, Mathers C, Hogan D (2016). Stillbirths: Rates, risk factors, and acceleration towards 2030. Lancet..

[2] Ashman AM, Brown LJ, Collins CE, Rollo ME, Rae KM (2017). Factors associated with effective nutrition interventions for pregnant indigenous women: a systematic review. J Acad Nutr Diet.

[3] Goicolea I (2001). Exploring women’s needs in an Amazon region of Ecuador. Reprod Health Matters.

[4] Gracey M, King M (2009). Indigenous health part 1: determinants and disease patterns. Lancet..

[5] Ramírez-Luzuriaga MJ, Belmont P, Waters WF, Freire WB (2019). Malnutrition inequalities in Ecuador: Differences by wealth, education level and ethnicity. Public Health Nutr.

[6] Mundo Indígena 2019: Ecuador - IWGIA - International Work Group for Indigenous Affairs. https://www.iwgia.org/es/ecuador/3396-mi2019-ecuador.html. Accessed 21 Oct 2020.

[7] INEC. Ecuador en cifras. 2021. https://www.ecuadorencifras.gob.ec/documentos/web-inec/Sitios/inec_salud/index.html. Accessed 21 Jan 2021.

[8] Aldulaimi S, Mora FE (2017). Family medicine - world perspective a primary care system to improve health care efficiency : lessons from Ecuador. J Am Board Fam Med.

[9] OPS. Perfil de Sistema de Salud: Ecuador, monitoreo y análisis de los procesos de cambio y reforma. Washington D.C.; 2008.

[10] Gea-Izquierdo ENP. Características socioculturales, demográficas y de salud pública de las nacionalidades indígenas del ecuador. Quito, Ecuador.; 2021.

[11] Oliver J (2013). Mujeres indígenas. J Chem Inf Model.

[12] Haddrill R, Jones GL, Mitchell CA, Anumba DOC (2014). Understanding delayed access to antenatal care: a qualitative interview study. BMC Pregnancy Childbirth.

[13] Jinga N, Mongwenyana C, Moolla A, Malete G, Onoya D (2019). Reasons for late presentation for antenatal care, healthcare providers’ perspective. BMC Health Serv Res.

[14] Sumankuuro J, Mahama MY, Crockett J, Wang S, Young J (2019). Narratives on why pregnant women delay seeking maternal health care during delivery and obstetric complications in rural Ghana. BMC Pregnancy Childbirth.

[15] Corbett CA, Callister LC (2012). Giving birth. The voices of Ecuadorian women. MCN Am J Matern Nurs.

[16] Paredes I, Hidalgo L, Chedraui P, Palma J, Eugenio J (2005). Factors associated with inadequate prenatal care in Ecuadorian women. Int J Gynecol Obstet.

[17] Goicolea I, San Sebastian M (2010). Unintended pregnancy in the amazon basin of Ecuador: A multilevel analysis. Int J Equity Health.

[18] Llamas A, Mayhew S (2018). “Five hundred years of medicine gone to waste”? Negotiating the implementation of an intercultural health policy in the Ecuadorian Andes. BMC Public Health.

[19] Györik SA, Brutsche MH (2004). Complementary and alternative medicine for bronchial asthma: Is there new evidence?. Curr Opin Pulm Med.

[20] Gallegos CA, Waters WF, Kuhlmann AS (2017). Discourse versus practice: are traditional practices and beliefs in pregnancy and childbirth included or excluded in the Ecuadorian health care system?. Int Health.

[21] Verdezoto N, Carpio-Arias F, Carpio-Arias V, Mackintosh N, Eslambolchilar P, Delgado V, et al. Indigenous women managing pregnancy complications in rural Ecuador barriers and opportunities to enhance antenatal care. In: NordiCHI ’20: Proceedings of the 11th Nordic Conference on Human-Computer Interaction: Shaping Experiences, Shaping Society. 2020. p. 1–9.

[22] Abrahams N, Jewkes R, Mvo Z (2001). Health care-seeking practices of pregnant women and the role of the midwife in Cape town, South Africa. J Midwifery Women’s Heal.

[23] Whitehead M, Dahlgren G (2007). Concepts and principles for tackling social inequities in health: levelling up Part 2.

[24] Arcaya MC, Arcaya AL, Subramanian SV (2015). Inequalities in health: definitions, concepts, and theories. Rev Panam Salud Publica.

[25] Hartmann M, Khosla R, Krishnan S, George A, Gruskin S, Amin A (2016). How are gender equality and human rights interventions included in sexual and reproductive health programmes and policies: a systematic review of existing research foci and gaps. PLoS One.

[26] Fernández-Sáez J, Ruiz-Cantero MT, Guijarro-Garví M, Carrasco-Portiño M, Roca-Pérez V, Chilet-Rosell E, et al. Looking twice at the gender equity index for public health impact. BMC Public Health. 2013;13. 10.1186/1471-2458-13-659 PMC3751633 23855520

[27] Jensen KLB, Temple-Smith MJ, Bilardi JE (2019). Health professionals’ roles and practices in supporting women experiencing miscarriage: a qualitative study. Aust New Zeal J Obstet Gynaecol.

[28] Safaei J (2012). Democracy, human rights and women’s health. Mens Sana Monogr.

[29] Vibha Pathak BJ, Kalra S (2013). Qualitative research. Perspect Clin Res.

[30] Etikan I (2016). Comparision of snowball sampling and sequential sampling technique. Biometrics Biostat Int J.

[31] Thomas DR (2006). A general inductive approach for analyzing qualitative evaluation data. Am J Eval.

[32] Attride-Stirling. (2001). Thematic networks: an analytic tool for qualitative research. Qual Res.

[33] Malo Serrano M, Malo Corral N (2014). Health reform in Ecuador: never again the right tohealth as privilege. Rev Peru Med Exp Salud Publica.

[34] Quezada N (1975). Creencias Tradicionales sobre embarazo y parto. An Antropol.

[35] Ikehara S, Kimura T, Kakigano A, Sato T, Iso H, Saito H (2019). Association between maternal alcohol consumption during pregnancy and risk of preterm delivery: the Japan Environment and Children’s Study. BJOG.

[36] Villar J, Ba H, Piaggio G, Lumbiganon P, Belizán JM, Farnot U (2001). Articles WHO antenatal care randomised trial for the evaluation of a new model of routine antenatal care. Lancet.

[37] Gil-González D, Carrasco-Portiño M, Ruiz MT (2006). Knowledge gaps in scientific literature on maternal mortality: a systematic review. Bull World Health Organ.

[38] Phoa KYN, Chedraui P, Pérez-López FR, Wendte JF, Ghiabi S, Vrijkotte T (2016). Perinatal outcome in singleton pregnancies complicated with preeclampsia and eclampsia in Ecuador. J Obstet Gynaecol.

[39] Feeley C, Thomson G, Downe S (2019). Caring for women making unconventional birth choices : a meta-ethnography exploring the views , attitudes , and experiences of midwives. Midwifery..

[40] Burcher P, Hospital Y (2016). Home birth of infants with anticipated congenital anomalies : a case study and ethical analysis of providers ’ obligations.

[41] Olsen O, Clausen JA (2014). Planned hospital birth versus planned birth. Cochrane Database Syst Rev.

[42] Mignolo, Walter and Walsh C. On decoloniality, concepts, analytics, praxis. Duke Unive. 2018.

[43] Myer L, Harrison A (2003). Why do women seek antenatal care late ? Perspectives from rural South Africa. Br Rep.

[44] Goicolea I, Wulff M, Sebastian MS, Öhman A. Adolescent pregnancies and girls’ sexual and reproductive rights in the amazon basin of Ecuador: an analysis of providers’ and policy makers’ discourses. BMC Int Health Hum Rights. 2010;10. 10.1186/1472-698X-10-12 PMC2889876 20525405

[45] Mah BL, Pringle KG, Weatherall L, Keogh L, Schumacher T, Eades S (2019). Pregnancy stress, healthy pregnancy and birth outcomes - The need for early preventative approaches in pregnant Australian Indigenous women: A prospective longitudinal cohort study. J Dev Orig Health Dis.

[46] Armenta-Paulino N, Vázquez MS, Bolúmar F (2019). Obstacles and opportunities for monitoring ethnicity-based inequalities in maternal health care: Lessons from Mexico. PLoS One.

[47] OHCHR | Declaration on the Elimination of Violence against Women. https://www.ohchr.org/en/professionalinterest/pages/violenceagainstwomen.aspx. Accessed 10 Aug 2020.

[48] Chee VA, Teran E, Hernandez I, Wright L, Izurieta R, Baldwin J (2019). Desculturizacion, urbanization and nutrition transitipn among urban Kichwas indigenous communities residing in the Andes highlands of Ecuador. Public Health Nutr.

[49] Khamishon R, Chen J, Ranatunge N, Wu Q, Downey N (2019). Use and perception of contraception among genders in Santo Domingo, Dominican Republic. Ann Glob Heal.

[50] Kaswa R, Rupesinghe GFD, Longo-Mbenza B (2018). Exploring the pregnant women’s perspective of late booking of antenatal care services at Mbekweni Health Centre in Eastern Cape, South Africa. Afr J Prim Heal Care Fam Med.

[51] Dako-Gyeke P, Aikins M, Aryeetey R, Mccough L, Adongo PB (2013). The influence of socio-cultural interpretations of pregnancy threats on health-seeking behavior among pregnant women in urban Accra, Ghana. BMC Pregnancy Childbirth.

[52] Rasch D, Bywater K. Health promotion in ecuador : a solution for a failing system. Health. 2014:916–25.

[53] Hajizadeh M, Hu M, Bombay A, Asada Y (2018). Socioeconomic inequalities in health among Indigenous peoples living off-reserve in Canada: trends and determinants. Health Policy.

[54] Okoli C, Hajizadeh M, Rahman MM, Khanam R (2020). Geographical and socioeconomic inequalities in the utilization of maternal healthcare services in Nigeria: 2003-2017. BMC Health Serv Res.

[55] Ruiz-Cantero M, Carrasco-Portiño M, M EF, C DS, de Sierra C. (2007). The myopia of governments contributes to maternal mortality: dying from socioeconomic and physical distances. J Epidemiol Community Health.

[56] Feld H, Ceballos Osorio J, Bahamonde M, Young T, Boada P, Rayens MK. Poverty and paternal education associated with infant safe sleep intentions in a Peri-Urban community in Ecuador. Glob Pediatr Heal. 2021;8. 10.1177/2333794X211044112 PMC8411618 34485625

[57] Franco Á, Álvarez-Dardet C, Ruiz MT (2004). Effect of democracy on health: ecological study. Br Med J.

[58] Ruiz-Cantero MT, Guijarro-Garvi M, Rose D, Martínez-riera JR, Fernández-sáez J (2019). Health & Place Governance commitment to reduce maternal mortality. A political determinant beyond the wealth of the countries. Health Place.

[59] Harfield S, Davy C, Kite E, McArthur A, Munn Z, Brown N (2015). Characteristics of Indigenous primary health care models of service delivery: a scoping review protocol. JBI Database System Rev Implement Rep.

